# Rubella outbreak investigation, Gokwe North District, Midlands province, Zimbabwe, 2014 - a case control study

**DOI:** 10.11604/pamj.2015.22.60.5939

**Published:** 2015-09-22

**Authors:** Annamercy Chenaimoyo Makoni, Milton Chemhuru, Donewell Bangure, Notion Tafara Gombe, Mufuta Tshimanga

**Affiliations:** 1 Department of Community Medicine, University of Zimbabwe, Zimbabwe; 2 Ministry of Health and Child Care, Zimbabwe

**Keywords:** Rubella outbreak, risk factors, Gokwe North

## Abstract

**Introduction:**

Rubella is a contagious disease, caused by rubella virus and transmitted via the respiratory route. Rubella in pregnancy may cause Congenital Rubella Syndrome (CRS), characterized by multiple defects to the brain, heart, eyes and ears. Gokwe North experienced an increase in rubella cases from 6 cases (24 June 2014) to 374 cases (12 August 2014). The study was conducted to determine risk factors associated with contracting rubella.

**Methods:**

A 1:1 unmatched case control study was conducted. A case was a child <15 years, resided in Gokwe North, with maculopapular rash and tested positive for rubella specific IgM or was linked epidemiologically to a laboratory confirmed case. Blood was collected for laboratory diagnosis. An interviewer administered questionnaire was used. Epi Info™ was used to analyze data.

**Results:**

Eighty eight cases and 88 controls were recruited, median age for cases was 7 years (Q1 = 4, Q3 = 8) and 6 years (Q1 = 3, Q3 = 9) for controls. Independent risk factors for contracting rubella were; classmate contact (AOR 9.44; (95% CI 4.29-20.77)) and having >3 children in a household (AOR 2.59; 95%CI (1.23-5.42)). Only 10.2% and 6.8% of the caregivers’ cases and controls respectively, knew rubella is spread through contact with an infected person (p = 0.57). Majority of caregivers (97.8%) reported to the health facility within two days of onset of rash.

**Conclusion:**

Outbreak was driven by contact at school and was spread into the community through school children. Screening and isolation of the sick controlled the outbreak. Routine rubella vaccination could be considered to prevent similar outbreaks.

## Introduction

Rubella is a viral disease which is caused by the rubella virus, an envelope, positive-stranded RNA virus (family Togaviridae, genus Rubivirus) [1]. It is a contagious disease, which is transmitted via the respiratory route. The average incubation period is 14 days, with a range of 7 to 21 days. Infection is up to five days after the onset of rash [2]. Rubella usually occurs in seasonal patterns, with epidemics every 5-9 years. Its public health importance is due mainly to the teratogenic potential of the virus [2]. Rubella is of greatest danger to the unborn fetus. Up to 90% of infants born to mothers infected in the first trimester will develop the physical anomalies referred to as congenital rubella syndrome (CRS) [1]. CRS is characterized by blindness, heart defects, deafness, behavioral disorders, mental retardation, growth retardation, bone disease, enlarged liver and spleen, thrombocytopenia, and purple skin lesions, making it of public importance. Rubella is transmitted from person to person by droplet or direct contact with the nasopharyngeal secretions of an infected person or with the nasopharyngeal secretions or urine of an infant with CRS [1]. Transplacental infection resulting in CRS occurs in infants who are born to women with rubella occurring at 20 weeks or less of gestation [1]. During the second week after exposure, prodromal illness which consist of fever <39oC, malaise and conjunctivitis occurs. Postauricular, occipital and posterior cervical lymphadenopathy is characteristic and typically precedes the rash by 5-10 days. The rash usually starts on the face and neck before progressing down the body lasting 1-3 days [2]. Globally, 121,344 cases of rubella were reported by the World Health Organization (WHO) in 2009 [1]. The WHO reported an increase in rubella cases in the African Region from 865 in 1990 to 17 388 in 2009 [1]. About 5% of rubella infections occur in women of child bearing age and 90% of these women pass the rubella infection to their babies [1]. Worldwide it is estimated that there are more than 100 000 infants born with rubella syndrome each year [3]. In infants, CRS can be detected in nearly 100% at the age of 0-5 months. Zimbabwe reported 28 rubella cases in 2009 and 447 cases in 2011. Although several rubella outbreaks are being experienced, the national burden of rubella is not well documented. Zimbabwe is taking its first steps in measuring the disease burden in order to plan for the introduction of the rubella vaccine. However Harare Central Hospital was selected by the Ministry of Health and Child Care to be a CRS sentinel surveillance site for the country, to identify children under the age of 12 years with suspected CRS and the burden would determine the need for the rubella vaccine [3]. Gokwe North, one of the eight districts in the Midlands province has a population of 253 506 people. It is made up of 36 administrative wards and composed of three settlement types which are communal land (29 wards), resettlement (4 wards), and subsistence farming (3 wards). According to the district reports, Gokwe North has experienced measles outbreaks and has never detected rubella before [4]. On the 24^th^ of June 2014, 6 cases of suspected measles were seen at Simchembu clinic in Gokwe North district. There was a sudden increase of cases from Simchembu 2 Primary School. It was a rubella outbreak following laboratory confirmation of cases. The Index case was a 9 year old girl from Simchembu 2 Primary School who had onset of rash on the 17^th^of June and continued attending school. The number of cases continued to increase; more cases were from a neighboring primary school and the community. By the 12^th^ of August, 374 rubella cases had been line listed. It is against this background that we sought out broadly to investigate the rubella outbreak in Gokwe North District, Midlands Province, Zimbabwe.

## Methods

A 1:1 unmatched case control study was conducted. According to the WHO rubella case definition, a case was a child aged less than 15 years who resided in Gokwe North district who presented at Simchembu clinic with maculopapular rash and tested positive for rubella specific IgM or was linked epidemiologically to a lab confirmed rubella case during the outbreak period. A control was a child less than 15 years who resided in the same community with no history of signs and symptoms of rubella. A contact was defined as an individual sharing a confined space in close proximity for a prolonged time period such as one hour, increasing the risk of exposure to secretions either on explosive sneeze or cough. We defined attack rate as the proportion of susceptible individuals exposed to rubella who became infected. Simple random sampling (lottery method) was conducted to recruit study participants from the line list. A pretested interviewer administered questionnaire was used to collect data from caregivers of cases and controls in the community. The pretest was done at the clinic and adjustments on the questionnaire were made, some questions were edited for clarity. Epi Info™ was used to generate frequencies, means and odds ratios and 95% confidence intervals. Stratified analysis was done to control for confounding and to assess effect modification. Forward step-wise logistic regression was used to determine independent risk factors for contracting rubella. Permission to carry out the study was obtained from the Provincial Medical Director (PMD) for Midlands Province, Health Studies Office (HSO) and Gokwe North District Medical Officer (DMO) and the local chiefs. Written informed consent was obtained from study participants.

## Results

### Descriptive epidemiology

**Figure 1:**
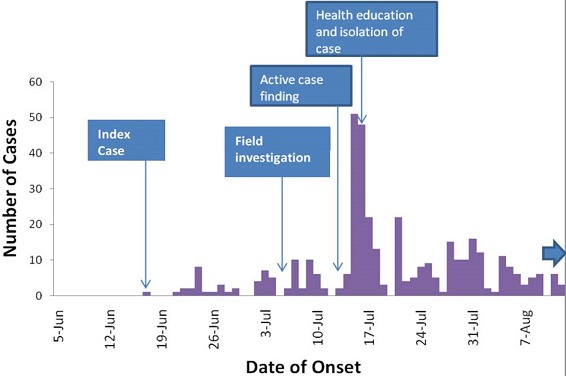
An epi curve of the rubella outbreak, Gokwe North District, Midlands Province, Zimbabwe, 2014

A total of 374 cases of rubella were seen between the 24^th^ of June and the 12^th^ of August 2014 at Simchembu clinic, Gokwe North district. Six cases were laboratory confirmed and the rest were epidemiologically confirmed cases. Of these, 235 (63%) were females and 139 (37%) were males, showing a female to male ratio of 1.7:1. One hundred and two (27%) cases were under fives, one hundred and seventy seven (47%) cases were children in the 5-9 years age group, eighty nine (24%) cases were above 10 years and 6 (2%) were women of child bearing age. Gokwe North district, Wards 1 and 31 were affected by the rubella outbreak. One hundred and sixty six (44%) cases were children from Simchembu 1 Primary School and one hundred and seventeen (31%) cases were from Simchembu 2 Primary School. The highest risk of developing rubella was at Simchembu 1 Primary School with an attack rate of 16.6%, followed by Simchembu 2 Primary School with an attack rate of 11.7%. Figure 1 illustrates the distribution of rubella cases with time at Simchembu Clinic, Gokwe North district. The epicurve shows several peaks suggesting a propagated outbreak. The outbreak was detected at the health facility level on the 24^th^ of June 2014. The District Health Executive (DHE) intervened on the 11^th^ of July. There was a sharp increase of cases to a peak of 51 cases on the 15^th^ of July. Cases steeply declined to zero cases on the 20^th^ of July and spiked to 22 cases on the 21^st^. There was a gradual decrease in number of cases from the 31^st^ of July until after the 12^th^ of August.

### Analytic epidemiology

A total of 88 cases and 88 controls of children below 15 years were recruited into the study. Of the 88 cases 53% were males and 47% were females. Of the 88 controls, 50% were males and the rest were females. The median age of cases was 6.5 years (Q_1_=4; Q_3_=8) and of the controls was 5.5 years (Q_1_=1; Q_3_=9). The majority of both cases and controls were of the age group 5-9 years, 53.4% and 45.5% respectively. The median age of care givers was 29 (Q_1_=24; Q_3_=37) for cases and 29(Q_1_=24.5; Q3 = 37.5) for the controls. Cases and controls were statistically comparable for age (p= 0.06), sex (p= 0.65) and religion (p= 0.28). However there was a statistically significant difference between cases’ and controls’ caregivers’ level of education (p = 0.002), (Table 1).

**Table 1:** Socio- demographic characteristics for cases, controls and caregivers, Gokwe North District, Midlands Province, 2014

Variable	Category	Cases n = 88 (%)	Controls n = 88 (%)	P value
**Age of Child**	<5 years	24 (27.3)	36 (40.9)	0.06
	5-9 years	47 (53.4)	40 (45.5)	
	10-15years	17 (19.3)	12 (13.6)	
**Sex of child**	Male	47 (53.4)	44 (50)	0.65
	Female	41 (46.6)	44 (50)	
**Age of Caregiver**	<25 years	23 (26)	22 (25)	0.65
	25-30	24 (27)	28 (31)	
	31-35	15 (17)	12 (14)	
	36-40	15 (17)	13 (15)	
	41+	11 (13)	13 (15)	
**Religion**	None	38 (43.2)	32 (36.4)	0.28
	Apostolic	33 (37.5)	35 (39.8)	
	Pentecostal	6 (6.8)	17 (19.3)	
	Orthodox	10 (11.4)	4 (4.5)	
	Traditional	1(1.1)	0 (0)	
**Caregiver Level of Education**	None	0 (0)	1 (1.1)	0.002
	Primary	15 (17)	32 (36.4)	
	Secondary	69 (78.4)	45 (51.1)	
	Tertiary	4 (4.5)	10 (11.4)	
**Median age of children (years)**	6.5 (Q_1_=4;Q_3_=8)	5.5(Q_1_=3;Q_3_=9)		
**Median age of caregivers (years)**	29(Q_1_=24;Q_3_=37)	29(Q_1_=25;Q_3_=38		

**Significant risk factors for contracting rubella:** statistically significant risk factors for contracting rubella in Gokwe North district were exposure to a classmate contact, (OR 7.31 (95%CI 3.68-48.49)), exposure to a household contact, (OR 3.32 (95%CI 1.65-6.69)), and having more than 3 children in a household (OR 2.35 ( 95%CI 1.27-4.33)) as shown in Table 2.

**Table 2:** Factors associated with contracting rubella in Gokwe North District, Midlands Province, 2014

Factor		Cases n = 88 (%)	Controls n = 88 (%)	OR	95% CI
**Household contact**	Yes	36 (40.9)	15 (17.2)	3.32^*^	[1.65-6.69]
	No	52 (59.1)	72 (82.8)		
**Neighbourhood contact**	Yes	8 (9.1)	6 (6.8)	1.37	[0.45-4.12]
	No	80 (90.9)	82 (95.2)		
**Classmate contact**	Yes	56 (63.6)	17 (19.3)	7.31^*^	[3.68-48.5]
	No	32 (36.4)	71 (80.7)		
**> 3 children in a household**	Yes	46 (52.3)	28 (31.8)	2.35^*^	[1.27-4.33]
	No	42 (47.7)	60 (68.2)		
**Sleeping in the same room**	Yes	53 (60.2)	46 (52.9)	1.35	[0.74-2.46]
	No	35 (39.8)	41 (47.1)		
**Suffered from rubella like illness before**	Yes	4 (4.54)	5(5.68)	0.79	[0.21-3.03]
	No	84 (95.46)	83 (94.32)		
**History of measles (monovalent) vaccination**	Yes	81 (92)	85 (96.6)	0.41	[0.10-1.63]
	No	7 (8)	3 (3.4)		

**Independent risk factors for contracting rubella**: independent risk factors for contracting rubella were exposure to a classmate contact (AOR 9.44 (95% CI 4.29-20.77)) and having more than three children within a household with a household contact (AOR 2.59 (95% CI 1.23-5.42)) (Table 3).

**Table 3:** Independent factors for contracting rubella in Gokwe North District, Midlands Province, 2014

Independent Factor	AOR	95% CI	P value
Classmate contact	9.44	[4.29-20.77]	<0.01
>3 children in a household	2.59	[1.23-5.42]	0.01

### Caregiver knowledge and perceptions on Rubella

Caregivers perceived the rubella illness as measles. All (100%) care givers perceived the illness as a dangerous disease and childhood immunization as important. Only 10.2% and 6.8% of the caregivers’ cases and controls respectively, knew that rubella is spread through contact with an infected person (p = 0.57). The majority of the caregivers (97.8%) reported to the health facility within two days of onset of rash and did not use any local herbs at home.

### Epidemic preparedness and response

The outbreak was detected at health facility level during week 26 on the 24^th^ of June 2014. The health facility conducted an investigation on the same day. The DHE intervened on the 11^th^ of July 2014. Active case finding was done in schools and in the community with the help of the Environmental Health Technician (EHT) and Village Health Workers (VHWs). Cases were screened, treated and quarantined. Cases were not allowed to mix with other children at home and at school. They were not allowed to play and sleep together with others. They were quarantined until after the infectious period, a week after onset of rash. Line lists were compiled daily. All cases were treated according to the national guidelines. Resourses were adequate for the outbreak response. Medicines were received from the district and from Medicines Sans Frontieres (MSF). The district availed 3 community nurses to the clinic and a doctor from MSF also joined the clinic staff. However, there were no Information Education and Communication materials (IEC) on rubella for community awareness and education.

## Discussion

The study sought to determine factors associated with contracting rubella in Gokwe North district using a case control study design. This study design has a potential for ascertainment bias, which was reduced by the use of case definitions. The epidemic curve has several peaks typical of a propagated outbreak, suggestive of person to person transmission. The index case which was also the primary case had onset of rash on the 17^th^ of June 2014 and was a daughter to the relief teacher who had recently joined the school from Masvingo, where he was reported to have been in contact with a “suspected measles” case. This might imply that the outbreak came from Masvingo and spread within the school then into the community through school children. Exposure to a classmate contact was an independent risk factor for contracting rubella. School children with rubella continued attending school during illness, thereby spreading the rubella infection to others in class through sneezing and coughing. This finding is therefore biologically plausible considering that rubella is spread through respiratory secretions. Similar findings with Mpeta et al, 2005 [5]. Household contact was a significant risk factor for contracting rubella in Gokwe North district. Children who contracted rubella from school were spreading the disease to their siblings at home. This implies that isolation at home could have stopped the spread of rubella to non school going children. This was consistent with the study done by Mpeta T et al in 2005 in Insiza District, Matebeleland Province and Muchedzi A et al in 2004 in Gweru district, Midlands Province where household contact was a significant risk factor for contracting rubella [6]. Having >3 children in a household was an independent significant risk factor for contracting rubella, thus overcrowding was highlighted to be the driver of the current outbreak. This implies that having more children in the household increased the risk of being in contact with the infected child. This was consistent with a study done in 1999 on rubella outbreak investigation by Danovaro-Holliday et al in the United States whereby overcrowding in the work place and at home was a risk factor for contracting rubella [7]. However, children continued to attend school thereby spreading the disease to other school children and community members, putting the pregnant mothers at risk. Having suffered from rubella like illness before was a protective factor to contracting rubella; this is consistent with the results in a study by Banda et al (2005) where history of rubella was a protective factor [8]. Rubella infection offers permanent immunity after natural infection. There could have been undetected rubella outbreaks before, thus the protective nature was identified but not significant in this study. Rubella vaccination plays a major role in the prevention of rubella by offering lifelong immunity; however Zimbabwe has not yet implemented rubella vaccination and plans are in place to implement it in 2015. Care givers perceived rubella as measles which are almost similar in terms of transmission and signs and symptoms and there was no significant difference in knowledge between cases and controls except for some signs and symptoms which were fever, cough and coryza where there was a significant difference. This might be due to recall bias whereby caregivers of cases were more likely to know how their children presented than of controls. However caregivers did not know the importance of quarantining sick children both at home and at school, thus the quick outbreak spread. The district responded timely to the outbreak, meetings were held daily at the health centre and line listing was done on a daily basis. However, there were no information, education and communication materials on rubella for community sensitization and this implies that community education was inadequate.

### Study limitations

There was a possibility that controls could have been infected with rubella virus but not yet developed signs and symptoms of rubella during the investigation period. This could have introduced ascertainment bias which might have reduced the strength of associations; the median age for the cases was 6.5 years and for the controls was 5.5 years because the school going children in the community were mostly affected than the non school going children; caregivers perceived the rubella illness as measles and they were knowledgeable on measles which has some similar risk factors and signs and symptoms with rubella; no published case control studies on rubella were available; hence the author used unpublished studies; the study was conducted on children who were 15 years and below, hence the results cannot be generalized to populations outside this age group.

## Conclusion

The primary school going children aged between 5 and 9 years were most affected by rubella in Gokwe North District, from the 17^th^ of June 2014 to the 12^th^ of August. The outbreak was driven by contact at school and was spread into the community through school children. Screening and isolation of pupils both at school and home controlled the spread of the outbreak. Health worker and community trainings on rubella were recommended. Routine rubella vaccination could be considered to prevent similar outbreaks.

### Public Health actions done

The following activities were done in order to control the rubella outbreak: sensitization of Health Workers, Village Health Workers, school teachers, school children and the community at large; active case finding in schools and in the community; isolation of cases both at school and in the community; health education sessions at the clinic, in schools and the community; feedback was given to the district and to the province.
